# Application of high-throughput sequencing in understanding human oral microbiome related with health and disease

**DOI:** 10.3389/fmicb.2014.00508

**Published:** 2014-10-13

**Authors:** Hui Chen, Wen Jiang

**Affiliations:** Department of Conservative Dentistry and Periodontics, Affiliated Hospital of Stomatology, College of Medicine, Zhejiang UniversityHangzhou, China

**Keywords:** oral microbiome, high-throughput sequencing, dental caries, periodontitis, apical periodontitis

## Abstract

The oral microbiome is one of most diversity habitat in the human body and they are closely related with oral health and disease. As the technique developing, high-throughput sequencing has become a popular approach applied for oral microbial analysis. Oral bacterial profiles have been studied to explore the relationship between microbial diversity and oral diseases such as caries and periodontal disease. This review describes the application of high-throughput sequencing for characterization of oral microbiota and analyzing the changes of the microbiome in the states of health or disease. Deep understanding the knowledge of microbiota will pave the way for more effective prevent dentistry and contribute to the development of personalized dental medicine.

## INTRODUCTION

Improvements in bio-technologies have spurred a large number of studies aimed at obtaining a better understanding of the composition and effect in microbiota and its associations with various human diseases. Early studies of the human microbiome have revealed most of the microbes that occupy in different habitats of human body and are ∼10 times more numerous than our own cells, more attention has paid to viewing ourselves as a supraorganism (Gill et al., 2006).

Traditional culture independent methods, such as DNA–DNA hybridization or cloning sequencing of DNA is widely used to identify oral organisms and found over 700 bacterial species in oral cavity (Aas et al., 2005). However, they still have significant biases that do not allow microbial diversity to be fully studied, as many low richness species cannot be detected.

Recently a major advance over conventional sequencing techniques is the development of high-throughput sequencing methods, which is a part of the next-generation sequencing (NGS) techniques (Ronaghi, 2001). These methods can largely be grouped into three main types: sequencing by synthesis (Roche 454 pyrosequencing, Illumina, The Ion Torrent system), sequencing by ligation (SOLiD, Polonator G.007 system), and single-molecule sequencing (Helicos, Pacific BioSciences). This has been proving amenable for use in massively parallel signature sequencing technologies (Rothberg and Leamon, 2008), including Roche 454 genome sequencers, Illumina sequencers, Applied Biosystems SOLiD sequencer, Life Technologies Ion Torrent, Helicos biosciences HeliScope, Pacific Biosciences SMRT DNA sequencer. The most frequently used methods are the 454 pyrosequencing (Roche, Bradford, CT, USA), Illumina (Illumina, San Diego, CA, USA), and SOLiD (Applied Biosystems, Foster City, CA, USA). Each of them had their own character. Nucleotide detection in Illumina and SOLiD systems is performed one at a time. As a result, homopolymer regions can be accurately sequenced. Second advantage of them is their high output per run compared to 454 pyrosequencing, which lead it soon became a workhorse for whole-genome resequencing applications and for exploring metabolic processing potential and pathway representation in health and disease. Owning to its optical signal decay and dephasing, these systems have relative short-read length (Zhou et al., 2010). On the other hand, the 454 pyrosequencing platform had long read length and relatively short run time. Furthermore it does not need to carry out an extra chemical deblocking step, which would reduce the chances of premature chain termination and non-simultaneous extension (Metzker, 2010; Zhou et al., 2010). Although the drawback of relatively high cost per megabase sequencing output, the 454 pyrosequencing has still become one of the most prevalent used worldwide (Rogers and Venter, 2005).

Oral cavity is one of the indispensable parts of the human microbiome habitat. The oral bacterial community dynamics is complicated and still far way to full understand. In this paper, we reviewed the human oral microbiome composite and its shifting which related with health and disease by application of high-throughput sequencing.

## THE CONCEPT OF HUMAN ORAL MICROBIOME

Oral microbiome, which is referred to as the oral microflora or oral microbiota, is defined as all the microorganisms residing in the human oral cavity and their collective genome. It was firstly coined by Lederberg and Mccray (2001) “to signify the ecological community of commensal, symbiotic, and pathogenic microorganisms that literally share our body space and have been all but ignored as determinants of health and disease.” The oral cavity harbors one of the most diverse microbiomes in the human body. And bacteria predominated in the oral cavity, while others are in relatively low proportions in the most circumstance.

Oral microbiome harbors on teeth, gingival sulcus, tongue, cheeks, hard and soft palates, and tonsils and it is a critical component of oral health and disease.

## THE DIVERSITY AND COMPOSITION OF ORAL MICROBIOME

Oral cavity, which is one of the largest and most complex human-associated microbial habitats, harbors large numbers of bacteria that can have important effects on health. During the past 40 years, a wealth of knowledge has been gathered about these bacteria: over 250 oral species have been isolated and characterized by cultivation, and over 450 species have been identified by culture-independent molecular approaches (Paster et al., 2006). By 454 pyrosequencing, we studied in 120 children at age 6 and found that there were about 2,000 phylotypes by clustering at 3% dissimilarity level in each sample of caries-active and caries-free children; and there were more phylotypes in saliva than in dental plaque samples from the same groups. At the genus level, sequences from saliva and plaque represented 203 different genera, while 153 different genera were found in dental plaque (120 genera in caries-active samples and 116 genera in caries-free samples) and 156 different genera were found in saliva (132 genera in caries-active samples and 115 genera in caries-free samples; Ling et al., 2010). No gender differences in oral microbiome diversity were detected. By using whole-metagenome sequencing approaches, Firmicutes, Actinobacteria, Bacteroidetes, Fusobacteria, and Proteobacteria were found account for 80–95% of the entire oral microbiome. At the genus level, a total of 58 distinct genera are present at an 0.1% abundance. The most abundant genera comprise previously characterized oral bacteria: *Actinomyces, Prevotella, Streptococcus, Fusobacterium, Leptotrichia, Corynebacterium, Veillonella, Rothia, Capnocytophaga, Selenomonas, Treponema*, and TM7 genera 1 and 5 (Liu et al., 2012).

### THE COMPOSITION OF ORAL MICROBIOTA IN HEALTH

Oral microbiome in 60 caries free children were analyzed with 454 pryosequencing in one of our studies (Ling et al., 2010). The result agreed with the other studies more and less, found that more than fourteen phyla, including Firmicutes, Bacteroidetes, Proteobacteria, Actinobacteria, Spirochaetes, and Fusobacteria, Euryarchaeota, Chlamydia, Chloroflexi, SR1, Synergistetes, Tenericutes, Cyanobacteria, OD2, and TM7 in healthy subjects (Zaura et al., 2009; Bik et al., 2010; Griffen et al., 2011). Among them, the vast majority (containing more than 80% of the taxa) of oral bacteria belong to Firmicutes, Bacteroidetes, Proteobacteria, Actinobacteria, Spirochaetes, and Fusobacteria (Ling et al., 2010). At genus level, over 200 genera were found in the oral microbiota. The most abundant genera include *Streptococcus, Prevotella, Neisseria, Haemophilus, Porphyromonas, Gemella, Rothia, Granulicatella, Fusobacterium, Actinomyces*, and *Veillonella*. At species level, it had been estimated that the number of species–level phylotypes were between 500 and 10000 (Keijser et al., 2008; Lazarevic et al., 2009), and each oral niches harbored 266 “species-level” phylotypes on average (Zaura et al., 2009). It is higher than the previously reported 10–81 species per site by using a 16S rRNA gene-based microarray (Preza et al., 2009). In Hasan et al.’s (2014) study, the microbiota of human saliva was analyzed by using the Illumina GAIIx and HiSeq 2000 instrument, and more than 175 bacterial species were found at >90% accuracy, including bacteria *Haemophilus influenzae, Neisseria meningitidis, Streptococcus pneumoniae*, and *Gammaproteobacteria*.

### THE CORE MICROBIOME IN HEALTH

In our study, there were 3530 OTUs (operational taxonomic unit) shared with the oral microbiota in each of the intact enamel surfaces of 60 children. Five phyla including Proteobacteria, Firmicutes, Actinobacteria, Fusobacteria, and Bacteroidetes were found in healthy individuals, while nine genera were common in all subjects, including *Actinomyces*,*Capnocytophaga, Corynebacterium, Derxia, Leptotrichia, Neisseria, Prevotella, Streptococcaceae Streptococcus*, and *Veillonella*. Thus, in agreement with other studies it was proposed that there might be a core microbiome in the oral environment (Shade and Handelsman, 2012; Jiang et al., 2014; Xu et al., 2014). The core microbiome is shared with most of individuals and comprised of the predominant species in healthy conditions of oral cavity (Zarco et al., 2012).

In Zaura’s study, 26% of the unique sequences, 47% of the OTUs were shared with oral microbiome in each sample of three healthy subjects. At the higher taxonomic levels, 72% of all taxa (genus level or above) were common to the oral microbiome of three adults, contributing to 99.8% of all reads. The result also suggested the existence of a core microbiome (Zaura et al., 2009). Moreover, Lazarevic et al. (2010) found salivary microbial community appeared to be stable at different time points (from 5 to 29 days), supported the concept of a core microbiome in health state. However, It is just the beginning of the understanding whether there is the core microbiome in different oral niches and needed more researches to keep verifying this concept.

### THE SITE-SPECIFICITY MICROBIOME

According to the Huse’ research, oral bacterial microbiota might be site-specificity and showed the different richness. Hard palate showed the lowest estimate of total richness, while the gingival plaque showed the highest estimate of total richness. The genus *Corynebacterium* had at least eight OTUs with five different profiles and colonized in different niches. For instance, *Corynebacterium matruchotii* was present almost exclusively in the supragingival plaque, while *Corynebacterium argentoratense* mostly in saliva and to a lesser extent on the hard palate (Huse et al., 2012). Further study also found similar result. It may due to the shedding of the epithelial cells and the shear forces from chewing in the buccal fold and the hard palate. Genera *Eubacterium* and *Prevotella* showed a significant association with the tongue dorsum. The papillary structure and the low redox potential of its surface might explain its significant site-specific bacterial association. *Lautropia mirabilis* was the only species significantly associated with the supragingival plaque, while *Treponema socranskii* was found only in the subgingival plaque (Preza et al., 2009). The anaerobic environment of subgingival plaque may explain their significant site-specific association. In the oropharynx, the distribution of Firmicutes, Proteobacteria, and Bacteroidetes was similar to that of saliva, and more Proteobacteria than that in the mouth (Lemon et al., 2010).

### THE MICROBIOME VARYING DURING DIFFERENT PERIODS OF AGE

The oral microbiota is various during different periods of age, which colonizes in the oral cavity and changes with the developmental status such as primary and permanent tooth. *Veillonella, Neisseria, Rothia, Haemophilus, Gemella, Granulicatella, Leptotrichia*, and *Fusobacterium* were predominant genera in infant samples, while *Haemophilus, Neisseria, Veillonella, Fusobacterium, Oribacterium, Rothia, Treponema*, and *Actinomyces* were present at higher levels in their parents. Saliva bacterial microbiome in adults had greater bacteria diversity than that in infants (Cephas et al., 2011). Crielaard et al. (2011) found a higher proportion of Proteobacteria (*Gammaproteobacteria, Moraxellaceae*) than that of Bacteroidetes in the deciduous dentition and Bacteroidetes (mainly genus *Prevotella*), *Veillonellaceae* family, Spirochaetes, and candidate division TM7 increased with increasing age. It may reflect variation of oral microbiome driven by biological changes with age (Crielaard et al., 2011). And by comparing salivary microbiota from healthy children and adults, it was found that the mean level of seven genera including *Moraxella, Leptotrichia, Peptostreptococcus, Eubacterium, Neisseriaceae, Flavobacteriaceae*, and SR1 were significant differences between children and adults in another research, implying the microbiome shifts during different ages (Ling et al., 2013).

## ORAL MICROBIOTA IN DENTAL CARIES

### THE ORAL MICROBIOTA IN CARIES

Dental caries is one of the most prevalent worldwide chronic infectious diseases (Petersen et al., 2005). The desire of a core theme in studying the characterization of the oral microbiota has being pursued to understanding the particular organisms with tooth decay in a way that implies causation. Most researches have suggested that *Streptococcus mutans* is the major pathogen of dental caries, for it is the most frequently detected bacteria in the caries lesions (Hamada and Slade, 1980; Loesche, 1986; Matee et al., 1992).

However, some recent studies indicate that the relationship between MS and caries is not absolute: high proportions of MS may persist on tooth surfaces without lesion development, and caries can develop in the absence of *Streptococcus mutans* (Bowden, 1997). And very recently, researches found that acidogenic and aciduric bacteria other than MS, are responsible for the initiation of caries (Kashket et al., 1996). As new pyosequencing technique applied in the oral microbiology, ever greater numbers of bacteria have been identified as being associated with caries. In our recent study, applying high-throughput barcoded pyrosequencing combined with PCR-denaturing gradient gel electrophoresis, around 120 genera were found in the oral microbiota of saliva and supragingival plaques from children aged 3–6 years old with and without dental caries. Our study showed that oral microbiota in children was far more diverse than previous studies reported and more than 200 genera belonging to 10 phyla were found in the oral cavity. Six genera (*Streptococcus, Veillonella, Actinomyces, Granulicatella, Leptotrichia,* and *Thiomonas*) were significantly different between caries-active and caries-free samples in plaque (Ling et al., 2010). Further research also found that three genera including *Streptococcus, Granulicatella,* and *Actinomyces* exhibited a relative higher abundance in severe early children caries subjects, whereas caries free subjects exhibited a relative higher abundance of *Aestuariimicrobium*, indicating that there might be no specific pathogens but rather pathogenic populations structure shafting would lead to the occurrence of dental caries (Jiang et al., 2013). Yang et al. (2012) found that caries microbiomes were significantly more variable. And 147 OTUs were associated with adults dental caries (Yang et al., 2012).

### THE MICROBIOTA SHIFTING IN THE DIFFERENT STAGE OF CARIES

Our further research also found that oral microbiota was specific at different stages of caries progression. Gomar-Vercher et al. (2014) collected 110 saliva samples from 12-year-old children and divided into six groups according to the International Caries Detection and Assessment System II criteria. They found that *Porphyromonas* and *Prevotella* showed an increasing percentage compared to healthy individuals and bacterial diversity diminished as the severity of the disease increased (Gomar-Vercher et al., 2014). We also studied microbiome of plaque from caries-active subjects in different caries stages including intact enamel, white spot lesions and carious dentin lesions by pyrosequencing technique. And the result showed that the diversity of the total plaque bacterial community in the health subjects were more complex than caries subjects, which is in accordance with Gomar-Vercher’s study. Moreover thirteen genera (including *Capnocytophaga, Fusobacterium, Porphyromonas, Abiotrophia, Comamonas, Tannerella, Eikenella, Paludibacter, Treponema, Actinobaculum, Stenotrophomonas, Aestuariimicrobium,* and *Peptococcus*) were associated with dental health, eight genera (including *Cryptobacterium, Lactobacillus, Megasphaera, Olsenella, Scardovia, Shuttleworthia, Cryptobacterium*, and *Streptococcus*) increased significantly in cavitated dentin lesions, and *Actinomyces* and *Corynebacterium* were present at significant high levels in white spot lesions, while *Flavobacterium, Neisseria, Bergeyella,* and *Derxia* were enriched in the intact surfaces of caries individuals (Jiang et al., 2014). Relatively high proportions of *Atopobium, Prevotella*, or *Propionibacterium* with *Streptococcus* or *Actinomyces* dominated in carious dentin lesions in Obata’s study (Obata et al., 2014).

## ORAL MICROBIOTA OF APICAL PERIODONTITIS

Apical periodontitis develops around the apex of the dental root and is caused primarily by root canal infection (Siqueira, 2001). Bacterial biofilm communities established in the apical part of infected root canals are conceivably of the most importance in the pathogenesis of apical periodontitis (Siqueira, 2002). For there was no strong evidence of the specific involvement of a single species with any particular sign or symptom of apical periodontitis been found with advancing technique. By using massive parallel pyrosequencing analysis, 187 bacterial species-level phylotypes, 84 genera and 10 phyla were found in the apical part of root canals of teeth with apical periodontitis. The most abundant and prevalent phyla were Proteobacteria, Firmicutes, Bacteroidetes, Fusobacteria, and Actinobacteria. And the mean number of species-level phylotypes per sample was 37. These results indicate that bacterial communities in apical periodontitis are more diverse than previously demonstrated (Siqueira et al., 2011). Another study investigated the microbial diversity in symptomatic and asymptomatic canals with primary endodontic infections by using GS FLX Titanium pyrosequencing. The result showed that the vast majority of sequences belonged to seven phyla including *Actinobacteria, Bacteroidetes, Firmicutes, Fusobacteria, Proteobacteria, Spirochetes*, and *Synergistetes*. And *Pyramidobacter, Streptococcus, Leptotrichia* constituted nearly 50% of microbial community in asymptomatic teeth, whereas *Neisseria, Propionibacterium*, and *Tessaracoccus* were frequently found in symptomatic teeth (Lim et al., 2011). Santos et al. (2011) performed barcoded multiplex pyrosequencing to compare the microbiota of dental root canal infections associated with acute or chronic apical periodontitis. They found that the most abundant phyla in acute infections were *Firmicutes, Fusobacteria,* and *Bacteroidetes*, while in chronic infections, the dominants were *Firmicutes, Bacteroidetes,* and *Actinobacteria*. And the most prevalent genera in acute infections were *Fusobacterium* and *Parvimonas* (Santos et al., 2011). In Hong’s report, the diversity of bacterial community profile of intracanal microbiota in primary and persistent endodontic infections associated with asymptomatic chronic apical periodontitis showed no significantly different. And Bacteroidetes was the most abundant phylum in both primary and persistent infections. Other reports also found Bacteroidetes was the most abundant phylum in both primary and persistent infections by using pyrosequencing (Hong et al., 2013). And in symptomatic periapical lesions, the most abundant phyla were Proteobacteria and Firmicutes, while the predominated genera were *Fusobacterium, Streptococcus, Prevotella, Corynebacterium, Porphyromonas,* and *Actinomyces* (Saber et al., 2012). Another research analyzed endodontic infections by deep coverage pyrosequencing and found that 179 bacterial genera in 13 phyla. Among them, Bacteroidetes was the most prevalent bacterial phylum (Li et al., 2010).

## ORAL MICROBIOTA IN PERIODONTITIS

Periodontitis is an inflammatory disease in which oral bacteria play an important role in the progress of disease. It is thought to be concerned to a polymicrobial etiology, and comprehensive studies were performed to elucidate differences of the complex communities between health and disease (Ashimoto et al., 1996). By comparing the periodontally healthy controls and subjects with chronic periodontitis, Griffen found that community diversity was higher than that in disease. 123 species were identified which were significantly more abundant in individuals with chronic periodontitis and 53 species were identified in health controls. Among them, Spirochaetes, Synergistetes, and Bacteroidetes were health-associated, whereas Proteobacteria, Clostridia, *Negativicutes* and *Erysipelotrichia* were associated with disease (Griffen et al., 2012). There are significantly different in abundance comparing the oral microbiome in deep (diseased) and shallow (healthy) sites by sequencing 16S rRNA genes. In the deep sites, 14 genus-level OTUs, including *Streptococcus, Actinomyces,* and *Veillonella*, were decreased, whereas 37 genus-level OTUs were present in increased abundance compared to shallow sites such as *Prevotella, Porphyromonas, Treponema,* and *Fusobacterium* (Ge et al., 2013). By utilizing pyrosequencing technique, the gram-negative genera *Selenomonas, Prevotella, Treponema, Tannerella, Haemophilus,* and *Catonella* are significantly enriched in periodontal disease, whereas a set of gram-positive genera are significantly enriched in healthy samples (*Streptococcus, Actinomyces,* and *Granulicatella*; Liu et al., 2012). Bacteroidetes was the most abundant phylum in samples of periodontal disease, whereas Actinobacteria and Proteobacteria were significantly increased in plaque of periodontal health in another metagenomic sequencing analysis. At genus level, microbial community of periodontal health were dominated by *Streptococcus, Haemophilus, Rothia,* and *Capnocytophaga*, while microbiota in periodontal disease exhibited high level of *Prevotella* (Wang et al., 2013). Another 16S rRNA gene sequencing analysis found that *Fusobacterium, Porphyromonas, Treponema, Filifactor, Eubacterium, Tannerella, Hallella, Parvimonas, Peptostreptococcus,* and *Catonella* showed higher relative abundances in the periodontitis group (Li et al., 2014).

By using an Ion Torrent Personal Genome Machine, the diversity of bacterial community increased after scaling and root planning therapy (SPR). The most striking difference was that periodontal pathogenic species including the genera *Porphyromonas, Tannerella, Treponema*, and *Filifactor* were removed only in the group treated with SPR and antibiotics (Jünemann et al., 2012). And the post-treatment plaque samples retained the highest similarity to pre-treatment samples of the same individual (Schwarzberg et al., 2014).

## CONCLUSION

The high through-put technique has largely expended our knowledge regarding the composition of the bacterial communities associated with healthy and disease. The oral microbiota is far more diverse than previous thought. And as a number of uncultivated organisms discovery, it has being shed light on the relationship between oral microbiota and the caries process and periodontitis developing and other oral disease. The new high-throughput methodologies are likely to approach our understanding of bacterial ecology in oral disease.

## Conflict of Interest Statement

The authors declare that the research was conducted in the absence of any commercial or financial relationships that could be construed as a potential conflict of interest.
